# Traffic Vehicle Counting in Jam Flow Conditions Using Low-Cost and Energy-Efficient Wireless Magnetic Sensors

**DOI:** 10.3390/s16111868

**Published:** 2016-11-06

**Authors:** Xu Bao, Haijian Li, Dongwei Xu, Limin Jia, Bin Ran, Jian Rong

**Affiliations:** 1 Key Laboratory for Traffic and Transportation Security of Jiangsu Province, Huaiyin Institute of Technology, Huai’an 223003, China; baoxu@hyit.edu.cn; 2 Beijing Key Laboratory of Traffic Engineering, Beijing University of Technology, Beijing 100124, China; jrong@bjut.edu.cn; 3 Department of Civil and Environmental Engineering, University of Wisconsin-Madison, Madison, WI 53706, USA; bran@wisc.edu; 4 College of Information Engineering, Zhejiang University of Technology, Hangzhou 310014, China; dongweixu@zjut.edu.cn; 5 State Key Laboratory of Rail Traffic Control and Safety, Beijing Jiaotong University, Beijing 100044, China; lmjia@bjtu.edu.cn

**Keywords:** traffic engineering, vehicle counting, jam flow, vehicle detection algorithm, wireless magnetic sensor

## Abstract

The jam flow condition is one of the main traffic states in traffic flow theory and the most difficult state for sectional traffic information acquisition. Since traffic information acquisition is the basis for the application of an intelligent transportation system, research on traffic vehicle counting methods for the jam flow conditions has been worthwhile. A low-cost and energy-efficient type of multi-function wireless traffic magnetic sensor was designed and developed. Several advantages of the traffic magnetic sensor are that it is suitable for large-scale deployment and time-sustainable detection for traffic information acquisition. Based on the traffic magnetic sensor, a basic vehicle detection algorithm (DWVDA) with less computational complexity was introduced for vehicle counting in low traffic volume conditions. To improve the detection performance in jam flow conditions with a “tailgating effect” between front vehicles and rear vehicles, an improved vehicle detection algorithm (SA-DWVDA) was proposed and applied in field traffic environments. By deploying traffic magnetic sensor nodes in field traffic scenarios, two field experiments were conducted to test and verify the DWVDA and the SA-DWVDA algorithms. The experimental results have shown that both DWVDA and the SA-DWVDA algorithms yield a satisfactory performance in low traffic volume conditions (scenario I) and both of their mean absolute percent errors are less than 1% in this scenario. However, for jam flow conditions with heavy traffic volumes (scenario II), the SA-DWVDA was proven to achieve better results, and the mean absolute percent error of the SA-DWVDA is 2.54% with corresponding results of the DWVDA 7.07%. The results conclude that the proposed SA-DWVDA can implement efficient and accurate vehicle detection in jam flow conditions and can be employed in field traffic environments.

## 1. Introduction

Information about a traffic state is an important precondition for traffic management, and control and traffic information service and guidance [1]. It is also the basis for making traffic safety strategies and detecting traffic accidents. Therefore, traffic information acquisition is a significant backbone problem for intelligent transportation systems (ITSs). Due to the application of ITSs in urban traffic systems [1,2,3], the demand for large-scale, all-weather, and all-day traffic information is critical. A prior solution of traffic information acquisition is the use of traffic sensors to realize vehicle counting. Traffic vehicle counting from fixed sensors determines the number of vehicles at specific locations and times in road networks. Accurate traffic vehicle counting is essential for the acquisition of other traffic information, such as traffic volumes, speed evaluation, and vehicle classification. Fixed traffic sensors have inherent advantages (all-weather, all-day, and fixed location) and are used for traffic information acquisition in many countries. In recent decades, many fixed devices have been proposed to obtain traffic information in the field of sensor technology, such as inductive loop detectors [4,5], infrared instruments [6], ultrasonic arrays [7], microwave radars [8], magnetic sensors [9,10,11,12,13], and video image processing systems [14,15]. The majority of these sensors are weather-dependent or pavement-unfriendly.

For the outstanding advantages and increasing usage scale of magnetic sensors, the ability to count traffic vehicles by magnetic sensors has gained considerable interest. Many studies explore magnetic sensor applications in vehicle counting and other types of traffic information acquisition. Magnetic sensors have several excellent advantages; they are low-cost, energy-efficient, small, wireless, and weather-independent sensors. Traffic surveillance by magnetic technology and corresponding wireless sensor networks have been proposed and implemented in related research areas, especially vehicle detection and counting [9,10,11,12,13,16,17,18,19,20], speed estimation [10,11,12], and vehicle classification [10,11,13,19,21,22,23,24].

Early in 1978, Marshall [9] proposed a magnetic-based vehicle detection method and analyzed some characteristics between magnetic fields and vehicles. Marshall noted that magnetic sensors may not be able to satisfy all requirements but should not be overlooked as a possible option for counting or sensing the presence of a vehicle. In 2007, Cheung and Varaiya [10] investigated a type of wireless sensor network with magnetic sensors as network nodes to implement traffic surveillance. They proposed an adaptive threshold detection algorithm (ATDA) to detect vehicles. This algorithm is suitable for addressing the drift problem that is caused by temperature or light. Their experimental results are satisfactory due to a detection accuracy of 99% for a dataset of 330 vehicles. Haoui et al. [11] introduced the vehicle detection system by Sensys Networks Inc. (Berkeley, CA, USA) and they deployed this system in arterials and freeways in several cities and states. In [11], a vehicle detection system is employed for vehicle counting and to collect vehicle occupancy and speed data. The accuracy of their system for vehicle counting and vehicle speed and occupancy data acquisition is comparable to the accuracy of well-tuned loops in different traffic congestion conditions. Sifuentes et al. [16] presented a vehicle detector with a magnetic and optical sensor that was intended as a sensor node for use with a wireless sensor network (WSN). The optical sensor is used to wake up a magnetic sensor. These two sensors, combined with energy-efficient event-based software, yielded a simple, compact, reliable, and low-energy sensor node for vehicle detection. Daubaras and Zilys [17] introduced a vehicle detection system with magnetic field sensors and showed that the method can detect stationary vehicles in a parking lot. Liepins and Severdaks [18] proposed a method for vehicle counting, which achieved a detection rate with an accuracy of 94.8%. The distinguishing feature of their method is the use of a type of noninvasive wireless magnetic sensor network to eliminate the need for inserting sensors into the road structure. Wang et al. [20] employed a type of wireless magnetic sensor network to detect vehicles in an urban environment. The magnetic sensors are deployed on the sides of roads. In this paper, the wavelets methods are employed to filter noise and improve the accuracies of the magnetic signals. To design two types of vehicle detection algorithms in their field experiments, the corresponding accuracy of each algorithm is 90.9% and 84.1%. However, one of the drawbacks noted in this paper is the small size of the vehicle sample.

However, minimal attention has been given to vehicle detection in jam traffic conditions, especially for stop-and-go movements, which are common in cities during peak hours. When vehicles drive at low speeds in jam flow conditions, magnetic waveforms will produce a “tailgating effect” because vehicles are located close to each other. A continuous vehicle signal will be mutually interfered by front and rear vehicles, which complicates the design of a high-performance vehicle detection algorithm. Thus, traffic vehicle counting in jam flow conditions will be a significant and practical topic. For low-speed congested traffic in large cities, Yang and Lei [19] proposed a fixed-threshold-state machine algorithm that is based on signal variance to detect vehicles within a single lane. The lack of vehicle samplings in their experiment is very effective for verifying their algorithm. Although the ATDA proposed in [10] solved the drift problem, it does not consider jam flow conditions, especially the “tailgating effect” that is caused by congested traffic, and no field experiments have been conducted in jam flow conditions based on the ATDA. The traffic state in jam flow conditions is the most complicated state, which hinders the design of an efficient and accurate vehicle detection algorithm that is based on magnetic sensors. Robust vehicle detection in jam flow conditions is challenging, especially for urban traffic.

This paper introduces a multi-function wireless traffic magnetic sensor (TrafficMS) and proposes an alternative, efficient, and accurate vehicle detection method to address the drift problem caused by weather environments and the “tailgating effect” caused by traffic flow environments. To achieve a low-cost, energy-efficient and easy installment, TrafficMS is suitable for large-area deployment and all-weather, all-day, traffic surveillance.

## 2. Design and Function of Low-Cost, Energy-Efficient and Wireless Magnetic Sensor

### 2.1. Principle of Magnetic Technology

The magneto-resistance effect is a phenomenon in which the electrical resistance of a conductor or semiconductor changes in different magnetic fields. The normal magneto-resistance effect is derived from the interaction of the magnetic field and the Lorentz force of electrons. The magnetic field will cause the deflection or spiral of the movement of current carriers, which causes an increasing collision probability of electrons and enlarges the electrical resistance of a conductor or semiconductor. Several types of magneto-resistance effects, such as ordinary magneto-resistance (OMR), giant magneto-resistance (GMR) and anisotropic magneto-resistance (AMR), have been observed, while AMR has been extensively applied. Iron, cobalt, nickel and their alloys, which are strong magnetic metals, are ferromagnetic metals. When the external magnetic field is parallel to the magnetization direction, the electrical resistance will not change with the external magnetic field. However, when the external magnetic field deviates from the magnetization direction, the electrical resistance will decrease. This phenomenon is the AMR effect of strong magnetic metals [25]. Since AMR sensors have the advantages of low energy, high sensitivity, small size, low noise, high reliability, and acceptable resistance to poor environments, they are increasingly employed in several application fields.

**Figure 1:**
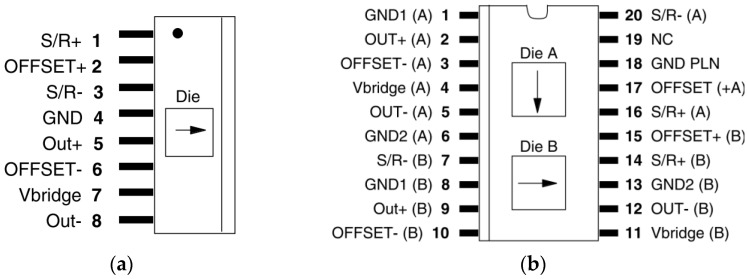
Pins of AMR sensors. (**a**) HMC1001 sensor; and (**b**) HMC1002 sensor.

The Honeywell HMC1001 and HMC1002 magnetic sensors are one-axis and two-axis surface mount sensors, respectively, which are designed for low-field magnetic sensing, which comprise a type of AMR sensor [26]. They are extremely sensitive, low-field, solid-state magnetic sensors that are designed to measure the direction and magnitude of Earth’s magnetic fields. The pins of HMC1001 and HMC1002 sensors are shown in Figure 1. HMC1001 is a single inline package (SIP) one-axis magnetic sensor whose sensing direction of a magnetic field is parallel to the pins; HMC1002 is a small outline-integrated circuit package (SOIC) two-axis magnetic sensor whose sensing directions are parallel and vertical to the pins. The change in the external magnetic fields will cause a change of voltage between OUT+ and OUT−, which reflects the strength of the external magnetic fields [26]. The on-chip current straps are included in HMC1001/HMC1002, and external coils are not needed, which renders them easy to use.

### 2.2. Magnetic Technology-Based Traffic Sensor

**Figure 2:**
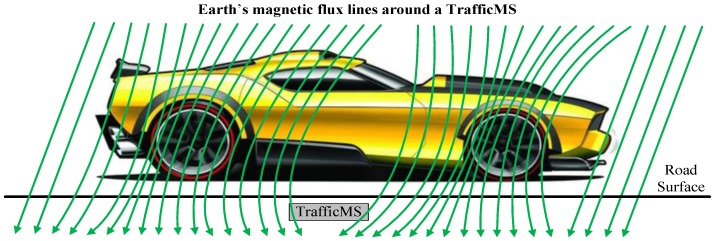
Disturbance of Earth’s magnetic flux lines by a vehicle.

The multi-function traffic wireless magnetic sensor (TrafficMS) was designed and developed by employing HMC1001/HMC1002 sensors. The TrafficMS can achieve the acquisition of several parameters of traffic flow, such as traffic volume, speed, headway and vehicle classification [12,27]. The sensing modules of the TrafficMS are HMC1001 and HMC1002 sensors, which can detect a miniscule change in magnetic field. Since vehicles contain ferromagnetic metal, the magnetic field around the TrafficMS will be disturbed when a vehicle passes a TrafficMS, as shown in Figure 2. This type of disturbance can be detected by HMC1001 and HMC1002 sensors and processed by an integrated circuit on TrafficMS. Then, the waveform data of the vehicle, which corresponds to the disturbance, will be recorded and outputted by TrafficMS.

By detecting the strength of a magnetic field in real-time, TrafficMS transfers the magnetic field signal to the voltage signal on an mV level and obtains the voltage signal on a V level using an amplifier. By reducing the noise signal with a filter, the TrafficMS outputs the corresponding digital value by an analog-to-digital converter (ADC). The digital value corresponds to the disturbance quantity of a magnetic field. When no vehicle exists around the TrafficMS, it will output a given digital value, which is referred to as the baseline *y*_b_ (no consideration of environmental noise). When a vehicle passes the TrafficMS, it will output waveform data with the baseline *y*_b_ and the disturbance value δ that is caused by the vehicle. The waveform data can be defined as the origin vehicle waveform data, which comprises the basis for achieving vehicle counting or other traffic information acquisition.

**Figure 3:**
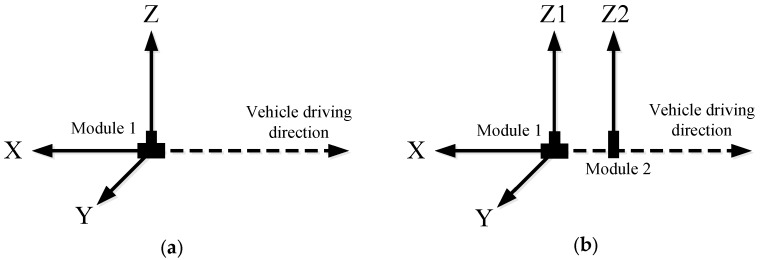
Sensing axes of the TrafficMS. (**a**) Single-module TrafficMS; and (**b**) double-module TrafficMS.

According to the detection and data demands, both single-module and dual-module TrafficMS units were designed and developed. Figure 3 shows the sensing axes of the TrafficMS. A single-module TrafficMS has a HMC1001 sensor and a HMC1002 sensor. The two sensors can sense the strength of the magnetic fields of three vertical axes (X/Y/Z-axis). A dual-module TrafficMS has two HMC1001 sensors and a HMC1002 sensor. The two HMC1001 sensors are located by a given spacing. A dual-module TrafficMS can sense the strength of the magnetic fields of three vertical axes (X/Y/Z1-axis) and one parallel axis (Z2-axis). In Figure 3, the X-axis points to the negative direction of running vehicles and reflects the disturbance of the magnetic field along the vehicle lane. The Y-axis points to the vertical direction of running vehicles and reflects the lateral disturbance. The Z-axis (Z1/Z2) points to the negative direction of gravity and records the presence and passing process of vehicles. The degree of sensitivity of each axis can be adjusted to adapt the valid sensing range for different applications.

Our previous study in [12] presented the dual-module TrafficMS, whose shape is a cylinder with an encircled diameter of 100 mm and a height of 65 mm. The shell material of TrafficMS is water-, dust-, and pressure-proof rigid plastic. TrafficMS adopts a series of chips (such as CC2430/CC2530) designed by Texas Instruments to achieve energy-efficient, low-rate, low-cost, and short-range wireless communication. In practice, several wireless communication protocols (such as SimpliciTI and Zigbee) can serve as an alternative embedded in the TrafficMS to construct WSNs. These WSNs can obtain traffic flow information in a regional road network.

**Figure 4:**
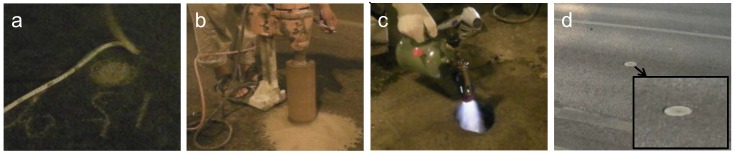
Quick installment of the TrafficMS. (**a**) Selecting a position; (**b**) drilling a hole; (**c**) improving the hole; and (**d**) deploying the TrafficMS and pasting the hole.

The installation of the TrafficMS is quick and convenient due to its circular shape and technical design. Figure 4 shows the process of a four-step quick installment of TrafficMS. The installation of TrafficMS requires approximately 10 min, which efficiently reduces the closing time of the vehicle lanes and the disturbance for normal traffic flow.

An outstanding feature of TrafficMS is the no-vehicle environment self-learning mechanism. After the TrafficMS is activated, the no-vehicle environment self-learning mechanism will measure and record the original strength of magnetic field when no vehicle appears around the TrafficMS. When a vehicle passes, the TrafficMS outputs the difference value of the current and the original strength of the magnetic field. This mechanism can ensure that the outputs of the TrafficMS will be less influenced by weather and geographical location factors. The frequency of no-vehicle environment self-learning can be determined by setting a self-learning period in the software (1 h, 30 min, and 10 min).

### 2.3. Advantages of TrafficMS

● Low-cost

As the reduction of chips and the development of integrated circuit, the low-cost, energy-efficient and high accuracy HMC1001/HMC1002 sensors and CC2430/CC2530 communication chips are used in the integrated design of TrafficMS. The shape of the TrafficMS is designed as a cylinder with an external diameter of only 100 mm and a water-, dust-, and press-proof rigid plastics.

● Environment self-learning mechanism

Through a no-vehicle environment self-learning mechanism, the TrafficMS can reduce the output errors caused by temperature drift and geographical position. Through a second traffic environment self-learning mechanism, the proposed vehicle detection algorithm can restrain the influence caused by the “tailgating effect”, especially under jam flow conditions. For sectional traffic vehicle counting, the case under jam flow conditions is the most difficult to counting vehicles accurately. These mechanisms makes it possible to obtain required detection results.

● Multi-functional detection

Using diversified detection modules and deploy strategies, the TrafficMS and the WSNs with TrafficMS nodes as network sensing nodes can achieve multi-parameter traffic information acquisition for the applications of traffic information service and control. A single TrafficMS node can detect or evaluate several kinds of traffic information such as traffic volume of a lane, individual vehicle speed, time headway, and vehicle classification. One or more WSN can acquire comprehensive traffic information, such as sectional traffic volume, average speed, density, occupancy, and vehicle classification proportion.

● Wireless communication and networking

Each TrafficMS has the function of wireless communication and is able to send its detection data to the roadside devices. For adopting a kind of energy-efficient, low-rate, and short-range wireless communication protocol (such as Simplici TI and Zigbee), the TrafficMS is able to have long-term, all-day detection. By networking technology with several TrafficMS nodes and roadside devices, WSNs to meet multiple traffic information detection demands can be built.

● Quick installment and replacement

Due to the low price and wireless communication feature, it is easy to install and replace a TrafficMS unit. It usually takes no more than 10 min to install a TrafficMS unit. When the power of the TrafficMS is used up, a new TrafficMS can be deployed near the original TrafficMS, and the only thing is to update the sensor ID of the new TrafficMS in the database.

## 3. Methods and Algorithms

The first step of traffic volume acquisition is single-lane vehicle detection. As a non-contact detection technology, magnetic technology has internal superiority in sectional traffic volume detection. However, the performance of a vehicle detection algorithm is related to the counting accuracy of the traffic volume, the complexity of computation, and the lifetime of a sensor node.

### 3.1. Basic Vehicle Detection Algorithm

**Figure 5:**
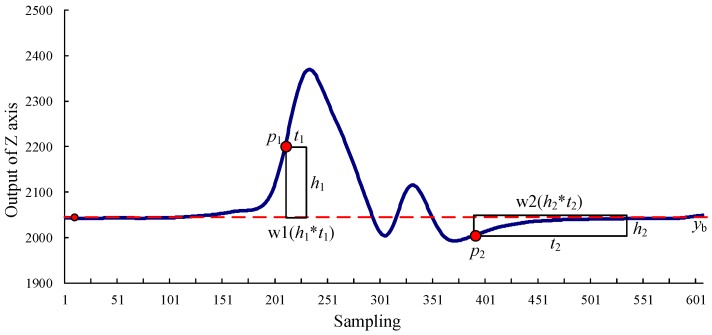
A vehicle waveform that is detected by the TrafficMS in free- or synchronized flow conditions.

A basic vehicle detection algorithm is applicable to conditions with a long distance between a front vehicle and a rear vehicle or a small “tailgating effect”, such as free-flow or synchronized flow conditions. Free-flow conditions always occur on a freeway, urban expressway, or urban roads with no influence by signalization. The speeds and headways are large in free-flow conditions, which have no “tailgating effect”. The traffic flow density of synchronized flow is larger and the speeds and headways are smaller than free-flow conditions. However, the “tailgating effect” can also be disregarded in synchronized flow conditions. In Figure 5, Li et al. [12] presented a vehicle waveform that is derived from the TrafficMS in free-flow conditions. Since the data of the Z-axis can reflect the presence and movement of a vehicle, only the data of the Z-axis will be the input of the vehicle detection algorithms in this paper, which may reduce their complexity. To eliminate the “tailgating effect”, the front and rear part of the vehicle waveform are almost coincident with the baseline *y*_b_. To adopt a no-vehicle environment self-learning mechanism to reduce or eliminate the effect of temperature or geographical position on the baseline, the baseline *y*_b_ is stable. According to the characteristics of the vehicle waveform in free-flow or synchronized flow conditions, the double window vehicle detection algorithm (DWVDA) (Algorithm 1) that is proposed in [12] is shown as follows:
**Algorithm 1.** Basic vehicle detection algorithm—DWVDA:  1: Initialization of the base line *y*_b_, the sampling frequency of the TrafficMS *f*_s_, the vehicle-approaching sensing window w1 (*h*_1_ × *t*_1_), and the vehicle-departing sensing window w2 (*h*_2_ × *t*_2_);  2: Start the vehicle-approaching sensing window w1;  3: Input the waveform data (the input data of sampling points are the width of w1 each time); if all sampling points are within w1, go to step 4 and denote the vehicle-approaching point as *p*_1_; otherwise, go to step 2;  4: Start the vehicle departing sensing window w2;  5: Input the waveform data (the input data of sampling points are the width of w2 each time); if all sampling points are within w2, denote the vehicle departing point as *p*_2_ and perform vehicle counting by adding 1. The waveform data between *p*_1_ and *p*_2_ is referred to as the vehicle waveform; detect the next vehicle. Otherwise, go to step 4.

The DWVDA can obtain the complete waveform of each vehicle and denote the approaching point and the departing point of each vehicle, which facilitates the acquisition of other traffic flow information, such as traffic volume, average speed, vehicle length, headway, and vehicle classification. The data from the Z-axis reflects the spatial disturbance of ferromagnetic material and records the presence and passing process of vehicles. In order to reduce data size and computation complexity, the DWVDA employs the data from the Z axis as the algorithm input. The algorithm has few parameters and a high computational efficiency. Therefore, it will be easily implemented in TrafficMS nodes with limited energy and computing capability to realize real-time and long-time vehicle detection.

### 3.2. Improved Vehicle Detection Algorithm for Jam Flow Conditions

**Figure 6:**
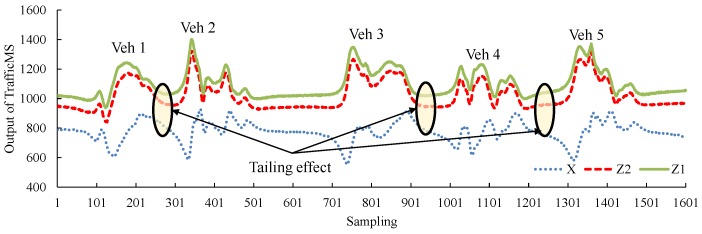
Waveforms of continuous traffic flow in jam flow conditions due to the tailgating effect.

The jam flow primarily exists on road segments and areas of congested freeways, ramps, toll facilities, urban expressways and signalized intersections. Especially during the peak hours of urban roads, the jam flow conditions are more common. In jam flow conditions, the density of traffic flow is large, the distance between a front vehicle and rear vehicle is small and the speed of traffic flow is low. Sometimes, vehicles will perform stop-and-go movements or even stop and queue. Figure 6 shows the vehicle waveforms of continuous traffic flows in jam flow conditions and the “tailgating effect” between two adjacent vehicles. When the TrafficMS detects a vehicle, the front vehicle may influence the magnetic field around the TrafficMS, which causes a weak performance of the DWVDA.

In traffic engineering, a common problem is low accuracy for vehicle counting by passive invasive traffic sensors (different from active non-invasive devices, such as RFID) in jam flow conditions. Martin et al. [28] investigated the performance of different sensor technologies that are influenced by some environmental and traffic state factors. According to the results of [28], the high volume state may be easier for influencing the performance of some sensor technologies than the low volume state.

Although the TrafficMS has a no-vehicle environment self-learning mechanism, in jam flow conditions the distance between a front vehicle and a rear vehicle is too small to be influenced by adjacent vehicles when a vehicle passes the TrafficMS. Then, the baseline *y*_b_ of the DWVDA will deviate from the origin value, which will yield a low accuracy. Due to changes of *y*_b_, the DWVDA with pre-set parameters will obtain only one vehicle when two or more vehicles pass the TrafficMS with a small spacing. A simple method is to minimize the width of w2. In this manner, the probability that two or more vehicles are identified as one vehicle will be reduced; however, the probability that one vehicle is identified as two or more vehicles will increase, especially for stop-and-go vehicles and large vehicles. Based on the DWVDA, a second adaptive DWVDA (SA-DWVDA) (Algorithm 2) was proposed to resolve this issue. Similar to the ATDA that is proposed in [10], the SA-DWVDA realized the second self-learning for traffic environment via an updated mechanism of the base line *y*_b_. The first self-learning mechanism causes TrafficMS to adapt a no-vehicle environment, whereas the second self-learning mechanism enables TrafficMS to adapt a traffic environment. The SA-DWVDA and its traffic environment update mechanism are shown as follows:
**Algorithm 2.** Improved vehicle detection algorithm for jam flow conditions—SA-DWVDA:  1: Initialization of *y*_b_, the sampling frequency of the TrafficMS *f*_s_, w1 (*h*_1_ × *t*_1_), w2 (*h*_2_ × *t*_2_), and the adjacent vehicle baseline *y*_b-veh_ = 0, the last baseline *y*_b-old_ = *y*_b_, the latest baseline *y*_b-new_ = *y*_b_, the second adaptive window w3 (*h*_3_ × *t*_3_), and the forgetting factor α.  2: Start the vehicle approaching sensing window w1 based on *y*_b-new_;  3: Input the waveform data (the input data of sampling points are the width of w1 each time); if all sampling points are within w1, go to step 4 and denote the vehicle approaching point as *p*_1_; otherwise, go to step 2;  4: Start the vehicle departing sensing window w2 based on *y*_b-new_;  5: Input the waveform data (the input data of sampling points are the width of w2 each time); if all sampling points are within w2, denote the vehicle departing point as *p*_2_ and perform vehicle counting is added by 1. If the waveform data between *p*_1_ and *p*_2_ is referred to as the vehicle waveform, go to step 6. Otherwise, go to step 4;  6: If all sampling points after point *p*_2_ are within w3, denote *y*_b-veh_ as the mean of all sampling points within w3 and go to step 7. Otherwise, go to step 2 to detect the next vehicle;  7: If |*y*_b-veh_-*y*_b-old_| < *d*_short_ and |*y*_b-veh_-*y*_b_| < *d*_long_, the update mechanism will begin; let *y*_b-old_ = *y*_b-new_ and *y*_b-new_ = (1 − α) *y*_b-old_ + *α* × *y*_b-veh_ and go to step 2; Otherwise, go to step 2 with no update operation.

**Figure 7:**
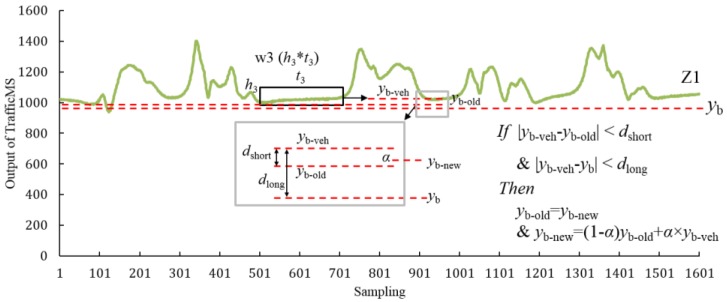
Parameters and their relationships of the SA-DWVDA.

Figure 7 shows the parameters and their relationships of the SA-DWVDA. w3 is the second adaptive window, which represents the minimal “tailgating” distance between the front vehicles and rear vehicles. If the waveform data do not satisfy the criteria of w3, the subsequent waveform data represents the same vehicle with previous waveform data. The parameter α is the forgetting factor. The larger is the value of α, the greater is the amount that the algorithm forgets and the lower is the weight of the old base line when calculating the new baseline. *d*_short_ is the short-term drift measure of *y*_b_ of the TrafficMS, which is the difference between the adjacent vehicle baseline and the last baseline. *d*_long_ is the long-term drift measure of *y*_b_, that is, the difference between the adjacent vehicle baseline and the baseline. When both values of *d*_short_ and *d*_long_ are within an established range, the update mechanism of the algorithm will be activated, which can improve the stability and robustness of the SA-DWVDA. The parameters of the SA-DWVDA can be calibrated based on field waveform data.

## 4. Experiment and Discussion

### 4.1. Field Experiments

**Figure 8:**
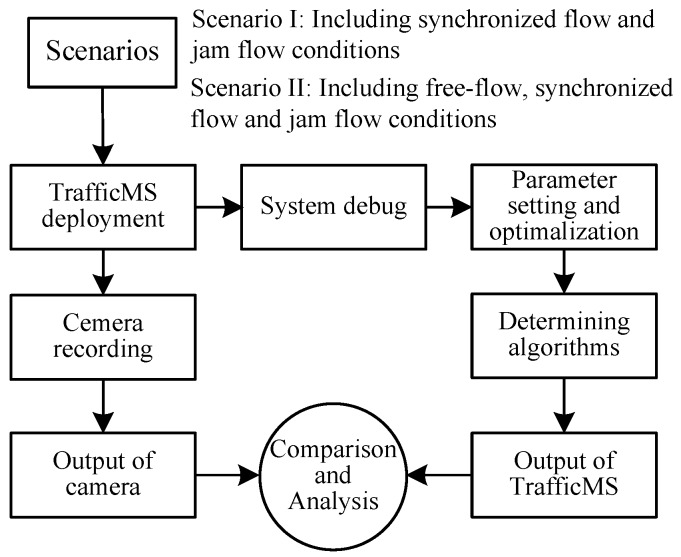
Experimental process.

To verify the detection performance of TrafficMS and the effectiveness of the proposed vehicle detection algorithms, the TrafficMS nodes were deployed in field traffic environments. These traffic scenarios include different traffic states of free-flow, synchronized flow, and jam flow conditions. The video cameras were used to record the actual traffic flow state, and the actual traffic volume data were obtained by manual statistics based on the video data that will be employed for comparison and analysis. Figure 8 shows the experimental process, which verifies the performance of different algorithms for the three traffic flow states.

For parameter setting and optimization, there are two main strategies. Strategy 1 is an empirical approach. Engineers give an initial value for each parameter and compare the results from TrafficMS and camera for a period time. Then, the engineers decide if it is necessary to adjust the parameter values according to detection results. For several-time adjustments, the TrafficMS may have a satisfactory or suboptimal result. Since each location of TrafficMS has a special traffic environment, strategy 1 is easy to implement, but sounds to be troublesome. Strategy 2 is a dynamic optimization approach. Engineers set a reasonable range for each variable parameter (excluding some constant parameters, such as *y*_b_, *f*_s_, and so on). TrafficMS collects the field traffic flow data for a period. The TrafficMS adjusts each parameter within its given range and outputs the detection result for each alternative combination. The alternative with the highest detection accuracy is selected as the final parameter setting. This paper adopts strategy 2 to implement dynamic parameter optimization. The final parameter values of the DWVDA and the SA-DWVDA are shown in Table 1.

(1) Scenario I: Including synchronized flow and jam flow conditions

In this scenario, TrafficMS was deployed in the center of a lane that belongs to a sub-arterial road in Beijing. TrafficMS can detect the real-time traffic state of this lane. This road is a two-lane road, which has no disturbances, such as lane changing among the vehicles on the road. The average speed of the vehicles on this road is a medium speed. The approximate distance between TrafficMS and the pedestrian signal is 30 m. When the signal is green, the synchronized flow will be the dominating mode; when the signal is red, the vehicles will queue and the dominating mode of traffic flow will transfer to jam flow. Several datasets with different time periods were obtained; both the DWVDA and the SA-DWVDA were tested based on these datasets. Table 2 shows the results of experiment scenario I for traffic vehicle counting. In Table 2, each dataset was named according to the starting time to be recorded, whose format is year-month-day-hour-minute.

As shown in Table 2, the performance of both the DWVDA and the SA-DWVDA is acceptable for vehicle counting in scenario I and the mean absolute percent error (MAPE) of their accuracies exceeds 99%. In the red signal interval, especially during the evening peak hour (such as the date set 200907291708), the mode of traffic flow will convert to the jam flow mode and the car-following distance between a front vehicle and a rear vehicle is very small (approximately 2 m). To reduce the disturbance of the “tailgating effect” between front and rear vehicles in jam flow conditions, the SA-DWVDA yields a better performance. For other conditions, the DWVDA and the SA-DWVDA can achieve satisfactory results. Although these datasets contain different climate and temperature conditions (summer and winter), different time periods (nonpeak hours and peak hours), different traffic signal conditions (smooth traffic during green signal intervals and queuing traffic during red signal intervals), the results are not significantly different under these conditions, which indicates that the algorithms of the DWVDA and the SA-DWVDA are robust.

In order to analyze the detailed counting-errors for individual vehicle, three possible errors are proposed and analyzed, which are “tailgating effect error” (TE), “big vehicle effect error” (BE), and “lane-changing error” (LE). In the algorithms of the DWVDA and the SA-DWVDA, the TE will reduce the counting number for detecting two or more adjacent vehicles as one vehicle; the BE will increase the counting number for detecting one large vehicle (such as bus, truck) as two or more vehicles; and the LE will lead to the manual statistical error using camera data which may increase or decrease the counting number for different visual angle of videos and different personal criterions. Table 3 shows the detailed counting-errors for individual vehicles of scenario I. For the road has no lane-changing disturbances, the value of the LE for each dataset is always 0. For scenario I, the performances of the DWVDA and the SA-DWVDA are similar, but the SA-DWVDA has a little advantage in dealing with the TE and the BE.

(2) Scenario II: Including free-flow, synchronized flow, and jam flow conditions

**Figure 9:**
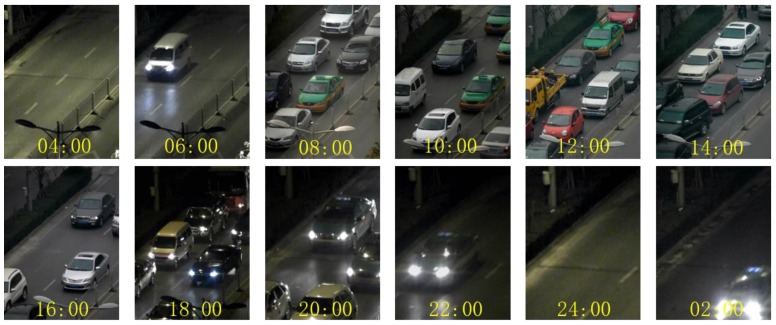
Field traffic flow states of experiment scenario II.

This scenario is located at a signalized intersection of an arterial in Xi’an, China. TrafficMS was deployed in the center of an outer straight lane near the signalized intersection. The traffic flow in the lane will be influenced by the traffic signal, which will cause a long vehicle queue. The vehicles in the traffic flow will exhibit car-following behaviors and stop-and-go movements due to the influence of heavy traffic. The algorithms of the DWVDA and the SA-DWVDA were tested based on the continuous vehicle waveform data that are derived from TrafficMS between 3 a.m. on 24 November 2012 and 3 a.m. on 25 November 2012. Figure 9 shows the traffic state within this 24-h period. Prior to dawn, the traffic volume was small and the free-flow mode was the dominating mode. Early in the morning as the traffic volume increased, the synchronized flow mode became the dominating mode. However, during the morning or evening peak hours, the traffic volume was high and the traffic congestion increased. Then, the phenomena of stop-and-go movements frequently occurred and the jam flow mode was the main mode.

Table 4 shows the results of experiment scenario II. Similar to scenario I, each dataset in Table 4 is named by the recording time from TrafficMS, which is year-month-day-hour-minute. Each dataset included an hour of continuous traffic flow data. The real hourly traffic volume on the lane located by TrafficMS was obtained by manual statistics based on continuous video data (the video data from 18:00 to 19:00 and 02:00 to 03:00 was incomplete; thus, the corresponding datasets were missing).

As shown in Table 4, the MAPE of the DWVDA for vehicle counting in a continuous 24-h period is 7.07%. By adopting the second adaptive mechanism, the SA-DWVDA reduces the MAPE to 2.54%; for any hour period, the accuracy of vehicle counting exceeds 90%. Generally, in free-flow conditions (such as datasets 201211240300, 201211240400, and 201211250100), the DWVDA and the SA-DWVDA have high accuracy, which is approximately 97%. However, in synchronized flow conditions (such as datasets 201211242100 and 201211242200), the accuracies of the DWVDA and the SA-DWVDA decrease; however, the results of the SA-DWVDA will improve. For jam flow, the traffic volume of a single lane with traffic signal control was more than 600, thus, the traffic flow speed was low and the car-following distance between front vehicles and rear vehicles was small. These phenomena triggered the “tailgating effect” (Figure 6) in the vehicle waveform data derived from the TrafficMS. In this case, the DWVDA could not eliminate the “tailgating effect” and the accuracy of the DWVDA decreased. The APEs of the DWVDA increased from 15% to 20% (refer to datasets 201211241300 and 201211241400). However, the SA-DWVDA was capable of solving this type of problem and reduce the counting errors. As shown in Table 4, in jam flow conditions, the hourly APEs of the SA-DWVDA were approximately 2%, which satisfied the vehicle counting demands for congested traffic. The experiment results indicate that the proposed vehicle counting methods compensate for the shortage of invasive sensors for vehicle counting.

Similarly, Table 5 shows the detailed counting-errors for individual vehicles of scenario II. As shown in Table 5, Scenario II has two lanes for each traffic direction. In addition, there are some lane-changing behaviors, especially during peak-hour periods. Except for LE, the TE and the BE are two main factors influencing algorithm performance. Compared with the DWVDA, the SA-DWVDA can reduce both the TEs and the BEs, especially in jam flow conditions and some synchronized flow conditions (from 201211240700 to 201211242000).

### 4.2. Analysis and Discussion

Based on the DWVDA that was proposed in [12], the SA-DWVDA proposed in this paper has achieved the continuous vehicle counting problem in jam flow conditions. Regarding the results of scenarios I and II, the detection results of scenario I are always better than the detection results of scenario II. One reason may be the time issue. If the time periods (predominantly 5–10 min) for the datasets in scenario I are shorter than the time periods for the datasets in scenario II, then the real traffic volumes in each dataset by the manual statistics will be more accurate. For an hourly period of each dataset, the statistic errors of real traffic volumes will be large. Another reason may be the traffic environment. A single lane in each direction is available in scenario I and the disturbance of lane changing will be weaker than scenario II. In scenario II, some vehicles change lanes near TrafficMS, which enlarges the errors in the vehicle waveform data and video data. In scenario II, the errors under synchronized flow conditions for some hours will be larger than the errors in jam flow conditions. The reasons may be related to the lane changing issue. The video demonstrates that the condition of lane changing is ideal in jam flow conditions and few behaviors of lane changing are observed. However, in synchronized flow conditions, the distance between a front vehicle and a rear vehicle will be large, which will increase the probability of lane changing and cause a large detection error.

In terms of algorithmic complexity, because the SA-DWVDA requires evaluation, the update criteria of the second adaptive traffic environment are dynamically evaluated, which may increase the complexity of the algorithm and the calculation of TrafficMS. For powering by dry batteries, to extend the lifetime of TrafficMS, an alternative scheme is to adopt a dual-algorithm in TrafficMS. In non-jam flow conditions, the DWVDA will be employed. In jam flow conditions, the SA-DWVDA will be activated. In engineering, a simple strategy is the use of the SA-DWVDA from 07:00 to 19:00 and the use of the DWVDA in other periods.

**Figure 10:**
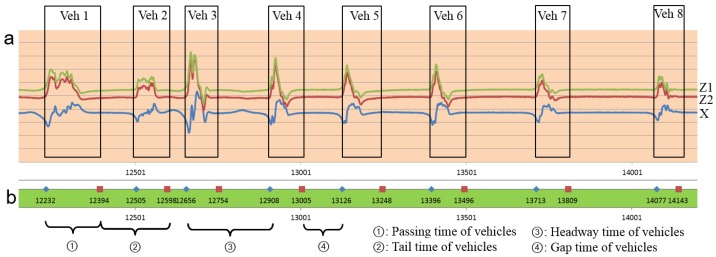
Some vehicle waveforms of dataset 201211240800 and the outputs of SA-DWVDA from 8:00 to 8:05. (**a**) Vehicle waveforms; and (**b**) outputs of SA-DWVDA.

Traffic vehicle counting is the basis of traffic information acquisition. Jam flow conditions are key and difficult points for traffic vehicle counting. To achieve accurate vehicle counting in jam flow conditions, a key step is to achieve entire space-time and networked traffic information acquisition. The accurate detection of time and state information when vehicles pass TrafficMS, facilitates the acquisition of other information (such as sectional traffic volumes, hourly traffic volumes, individual vehicle speed, headway, time or space occupancy) in many practical applications of ITS. The outputs of the DWVDA and the SA-DWVDA that are proposed in this paper not only show the traffic volume of any period but also provide the approaching time and departing time when a vehicle passes the TrafficMS, which facilitates the implementation of multi-parameter sensing of traffic flow. Figure 10 shows some vehicle waveforms for dataset 201211240800 and the outputs of the SA-DWVDA from 8:00 to 8:05 (a continuous traffic flow with eight vehicles). In Figure 10, a diamond represents the approaching time when a vehicle passes TrafficMS and a square represents the departing time. From left to the right, the time interval of two adjacent diamonds is the headway between two vehicles. Similarly, the time interval between two adjacent squares is the tail time of the vehicles; the time interval between an adjacent diamond and square is the gap time between the vehicles; and the time interval between an adjacent square and diamond is the passing time between two vehicles.

## 5. Conclusions and Application Prospects

By considering the actual demands of large-scale and networked traffic information acquisition and the difficult points of vehicle counting in jam flow conditions, the paper proposed a vehicle detection and counting method based on magnetic technology. A low-cost, energy-efficient, and small-sized wireless traffic magnetic sensor was designed and developed. By investigating the car-following behaviors and “tailgating effect” in jam flow conditions, based on the DWVDA, an improved algorithm—SA-DWVDA—was proposed to solve the accuracy problem for vehicle counting in jam flow conditions. The SA-DWVDA adopted a second adaptive traffic environment mechanism and was tested and verified in two real traffic scenarios that contained free-flow, synchronized flow, and jam flow conditions. Compared with the DWVDA, the SA-DWVDA can achieve a high accuracy of vehicle counting not only in free-flow and synchronized flow conditions, but also in jam flow conditions. The experimental results also verified the advantages of magnetic technology for vehicle detection in terms of installment and maintenance costs, weather environment, wireless communication, and all-day operation. The feasibility of TrafficMS was also proven for the acquisition of several types of traffic information.

For the limitation of experiment sites and project investments, the traffic volume of a single lane was tested in experimental scenarios based on TrafficMS. The following study can be divided into three directions.

(1) Traffic vehicle detection experiments are performed for multi-lane and multi-section scenarios. Sectional traffic volume is the basis for traffic flow state evaluation and prediction. Therefore, the verification of the proposed method in multi-lane and multi-section scenarios will be more practical and meaningful.
(2) Traffic vehicle detection experiments for several scenarios. By deploying TrafficMS nodes for freeways, ramps, or urban expressways and obtain long-time, continuous traffic flow data to test the accuracy of TrafficMS in different traffic flow states (free-flow, synchronized flow and jam flow), data and scenario support for large-scale and networked applications of TrafficMS are necessary.
(3) WSN-based traffic vehicle detection experiments. The WSNs can be constructed by deploying several TrafficMS nodes and other communication devices in a detection area. Based on the WSNs and the algorithms proposed in this paper, an investigation of the detection and evaluation methods for several traffic flow parameters will be useful for some practical applications of traffic information services and traffic route guidance.

## Figures and Tables

**Table 1:** Final parameter values of DWVDA and SA-DWVDA.

**DWVDA**										
*y*_b_	*f*_s_	w1	w2							
980	100	40 × 10	30 × 35							
**SA-DWVDA**										
*y*_b_	*f*_s_	w1	w2	*y*_b-veh_	*y*_b-old_	*y*_b-new_	w3	*d*_short_	*d*_long_	α
980	100	40 × 10	30 × 35	0	980	980	40 × 100	20	30	0.1

**Table 2:** Results of experiment scenario I for traffic vehicle counting.

Data Sets	Dominant Mode	Counting from Camera (Vehs)	Output of DWVDA (Vehs)	Output of SA-DWVDA (Vehs)	APE of DWVDA (%)	APE of SA-DWVDA (%)
200905211118	Synchronized flow	45	45	45	0.00	0.00
200905211336	Synchronized flow	206	207	206	0.49	0.49
200906301726	Jam flow	104	104	104	0.00	0.00
200907291708	Synchronized flow	59	58	59	1.69	0.00
200907291714	Jam flow	24	24	24	0.00	0.00
200908081012	Synchronized flow	32	32	32	0.00	0.00
200908091114	Synchronized flow	37	37	37	0.00	0.00
200910191706	Jam flow	67	68	68	1.49	1.49
200910221600	Synchronized flow	55	54	55	1.82	0.00
200911120918	Synchronized flow	32	33	32	3.13	0.00
200911191000	Synchronized flow	57	58	58	1.75	1.75
200911191006	Synchronized flow	67	67	67	0.00	0.00
Total/MAPE		785	787	786	0.86	0.31

**Table 3:** Detailed counting-errors for individual vehicles of scenario I.

Data Sets	Counting from Camera (Vehs)	Detailed Counting-Errors of DWVDA (Vehs)	Detailed Counting-Errors of SA-DWVDA Vehs
200905211118	45	TE(0), BE(0), LE(0)	TE(0), BE(0), LE(0)
200905211336	206	TE(0), BE(+1), LE(0)	TE(0), BE(0), LE(0)
200906301726	104	TE(−1), BE(+1), LE(0)	TE(0), BE(0), LE(0)
200907291708	59	TE(−1), BE(0), LE(0)	TE(0), BE(0), LE(0)
200907291714	24	TE(0), BE(0), LE(0)	TE(0), BE(0), LE(0)
200908081012	32	TE(0), BE(0), LE(0)	TE(0), BE(0), LE(0)
200908091114	37	TE(0), BE(0), LE(0)	TE(0), BE(0), LE(0)
200910191706	67	TE(−1), BE(0), LE(0)	TE(−1), BE(0), LE(0)
200910221600	55	TE(−1), BE(0), LE(0)	TE(0), BE(0), LE(0)
200911120918	32	TE(0), BE(+1), LE(0)	TE(0), BE(0), LE(0)
200911191000	57	TE(−1), BE(+2), LE(0)	TE(0), BE(+1), LE(0)
200911191006	67	TE(0), BE(0), LE(0)	TE(0), BE(0), LE(0)

**Table 4:** Results of experiment scenario II.

Data Sets	Dominant Mode	Counting from Camera (Vehs)	Output of DWVDA (Vehs)	Output of SA-DWVDA (Vehs)	APE of DWVDA (%)	APE of SA-DWVDA (%)
201211240300	Free flow	88	89	85	1.14	3.41
201211240400	Free flow	98	96	97	2.04	1.02
201211240500	Free flow	109	114	112	4.59	2.75
201211240600	Synchronized flow	215	236	233	9.77	8.37
201211240700	Synchronized flow	541	599	583	10.72	7.76
201211240800	Jam flow	663	658	653	0.75	1.51
201211240900	Jam flow	772	723	755	6.35	2.20
201211241000	Jam flow	726	711	727	2.07	0.14
201211241100	Jam flow	743	677	727	8.88	2.15
201211241200	Jam flow	739	651	720	11.91	2.57
201211241300	Jam flow	784	665	773	15.18	1.40
201211241400	Jam flow	713	562	720	21.18	0.98
201211241500	Jam flow	721	632	720	12.34	0.14
201211241600	Jam flow	721	629	735	12.76	1.94
201211241700	Jam flow	642	594	689	7.48	7.32
201211241900	Jam flow	700	625	691	10.71	1.29
201211242000	Synchronized flow	616	632	619	2.60	0.49
201211242100	Synchronized flow	547	563	553	2.93	1.10
201211242200	Synchronized flow	433	463	456	6.93	5.31
201211242300	Synchronized flow	345	357	350	3.48	1.45
201211250000	Free flow	248	245	243	1.21	2.02
201211250100	Free flow	189	188	188	0.53	0.53
Total/MAPE		11,467	10,838	11,557	7.07	2.54

**Table 5:** Detailed counting-errors for individual vehicles of scenario II.

Data Sets	Counting from Camera (Vehs)	Detailed Counting-Errors of DWVDA (Vehs)	Detailed Counting-Errors of SA-DWVDA Vehs
201211240300	88	TE(−2), BE(+4), LE(−1)	TE(−2), BE(0), LE(−1)
201211240400	98	TE(−3), BE(+1), LE(0)	TE(−1), BE(0), LE(0)
201211240500	109	TE(−1), BE(+4), LE(+2)	TE(0), BE(+1), LE(+2)
201211240600	215	TE(−4), BE(+16), LE(+9)	TE(−2), BE(+11), LE(+9)
201211240700	541	TE(−16), BE(+60), LE(+14)	TE(−2), BE(+30), LE(+14)
201211240800	663	TE(−10), BE(+8), LE(−3)	TE(−8), BE(+1), LE(−3)
201211240900	772	TE(−45), BE(+1), LE(−5)	TE(−12), BE(0), LE(−5)
201211241000	726	TE(−18), BE(+3), LE(0)	TE(−1), BE(+2), LE(0)
201211241100	743	TE(−66), BE(+6), LE(−6)	TE(−10), BE(0), LE(−6)
201211241200	739	TE(−94), BE(+4), LE(+2)	TE(−22), BE(+1), LE(+2)
201211241300	784	TE(−117), BE(+2), LE(−4)	TE(−8), BE(+1), LE(−4)
201211241400	713	TE(−157), BE(+4), LE(+2)	TE(−1), BE(+6), LE(+2)
201211241500	721	TE(−89), BE(+2), LE(−2)	TE(0), BE(+1), LE(−2)
201211241600	721	TE(−111), BE(+11), LE(+7)	TE(−1), BE(+7), LE(+7)
201211241700	642	TE(−99), BE(+35), LE(+16)	TE(−2), BE(+41), LE(+16)
201211241900	700	TE(−72), BE(+1), LE(−4)	TE(−6), BE(+1), LE(−4)
201211242000	616	TE(−12), BE(+26), LE(+2)	TE(−1), BE(+2), LE(+2)
201211242100	547	TE(−2), BE(+16), LE(+2)	TE(−2), BE(+6), LE(+2)
201211242200	433	TE(−5), BE(+32), LE(+3)	TE(−1), BE(+21), LE(+3)
201211242300	345	TE(−5), BE(+17), LE(0)	TE(−2), BE(+7), LE(0)
201211250000	248	TE(−4), BE(+3), LE(−2)	TE(−3), BE(0), LE(−2)
201211250100	189	TE(−3), BE(+2), LE(0)	TE(−1), BE(0), LE(0)
